# Supplementary material for: Molybdenum’s Role as an Essential Element in Enzymes Catabolizing Redox Reactions: A Review
Source: Biomolecules. 2024 Jul 19;14(7):869. doi: 10.3390/biom14070869 (PMC11275037; doi:10.3390/biom14070869)
Supplement: Supplementary file 1 [file biomolecules-14-00869-s001.zip › supplementary caption.pdf]

Figure S1. 3D structure of bovine xanthine oxidase (XO), protein cleaved form.

Figure S2. 3D structure of human aldehyde oxidase (AO) protein.

Figure S3. 3D structure of recombinant chicken sulfite oxidase (SOX) protein at resting state.

Figure S4. 3D structure of mitochondrial amidoxime-reducing component 1 (mARC1) protein.
