# Supplementary figures and images for: Molybdenum’s Role as an Essential Element in Enzymes Catabolizing Redox Reactions: A Review
Source: Biomolecules. 2024 Jul 19;14(7):869. doi: 10.3390/biom14070869 (PMC11275037; doi:10.3390/biom14070869)

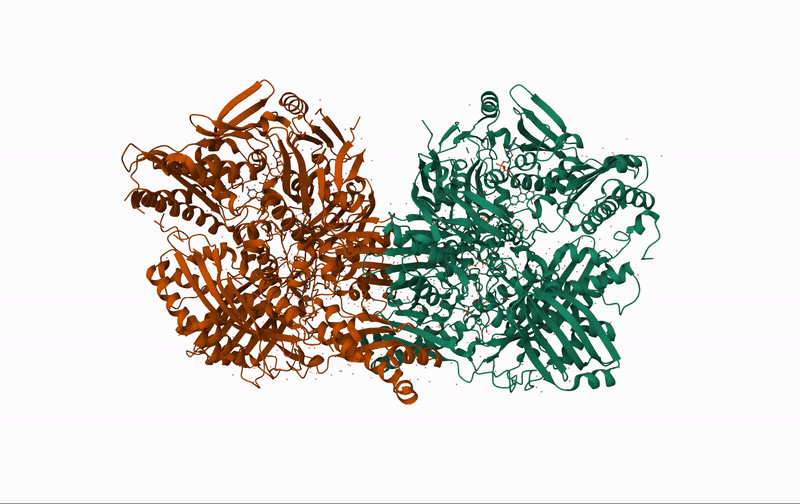

Supplement: Supplementary file 1 [file biomolecules-14-00869-s001.zip › Figure S1.gif]

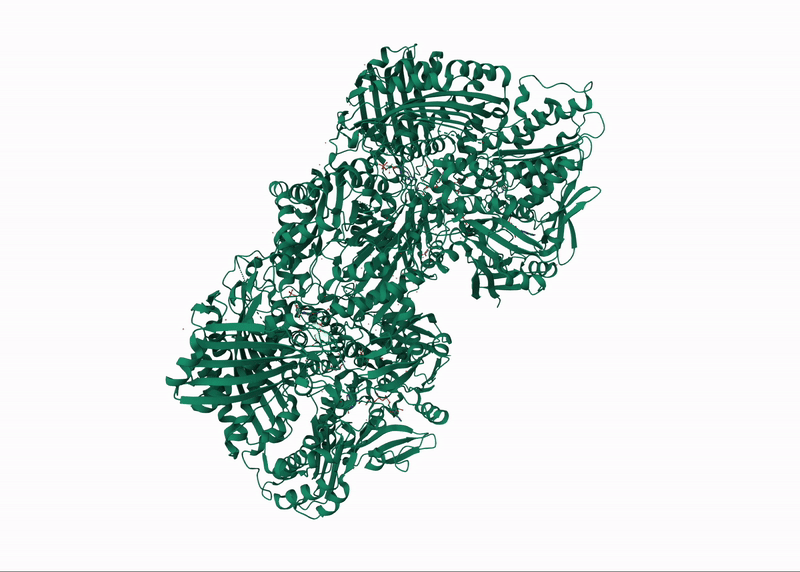

Supplement: Supplementary file 1 [file biomolecules-14-00869-s001.zip › Figure S2.gif]

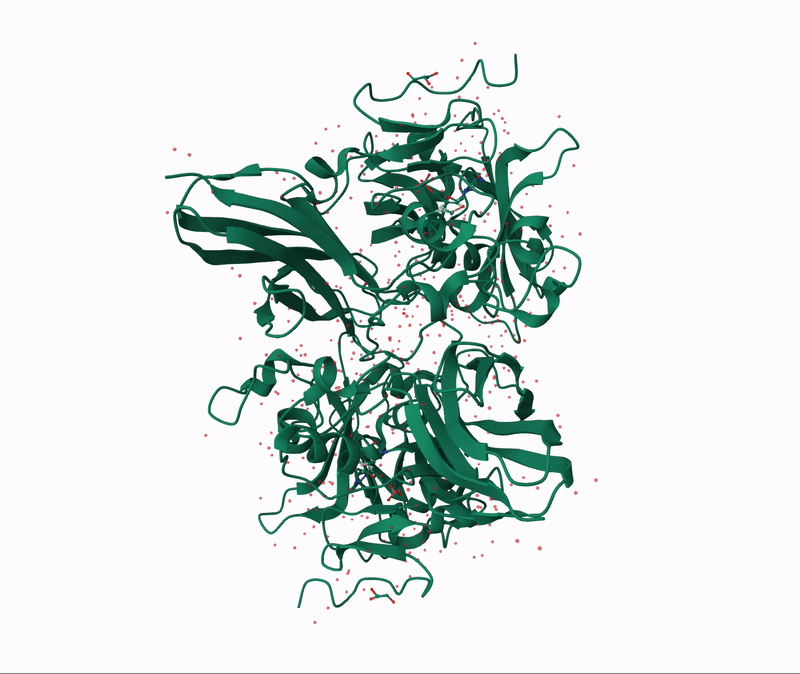

Supplement: Supplementary file 1 [file biomolecules-14-00869-s001.zip › Figure S3.gif]

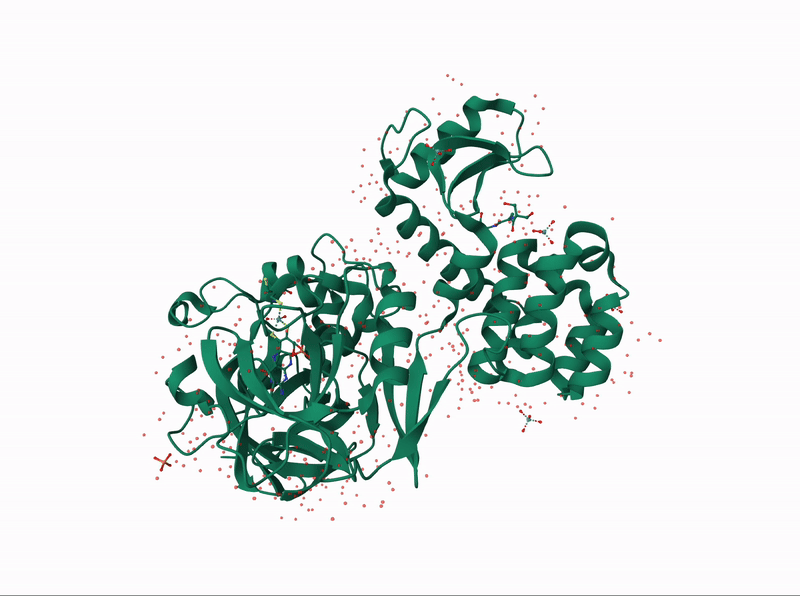

Supplement: Supplementary file 1 [file biomolecules-14-00869-s001.zip › Figure S4.gif]
